# Medical didactics during the pandemic: the asynchronous online seminar “Written Examinations” of the Frankfurter Arbeitsstelle für Medizindidaktik

**DOI:** 10.3205/zma001414

**Published:** 2021-01-28

**Authors:** Thomas Kollewe, Falk Ochsendorf

**Affiliations:** 1 Goethe-Universität Frankfurt, Fachbereich Medizin, Frankfurter Arbeitsstelle für Medizindidaktik, Frankfurt/Main, Germany; 2 Universitätsklinikum Frankfurt, Klinik für Dermatologie, Venerologie und Allergologie, Frankfurt/Main, Germany

**Keywords:** teacher training, online learning

## Abstract

**Objective: **Due to the prohibition of face-to-face courses during the Corona pandemic, the seminar "Written Examinations" of the Frankfurter Arbeitsstelle für Medizindidaktik (FAM) was converted into an asynchronous online seminar. This pilot project investigated how such a format is accepted and evaluated by the participants.

**Methodology: **A forum-based online format with group and individual tasks was chosen, which was didactically designed according to the problem-oriented design by Reinmann and Mandl.

**Results: **The seminar was attended by 14 people, 13 of whom took part in the evaluation. The overall evaluation was, with one exception, a grade of 2 (and better). The three items "practical relevance", "subjective learning success" and the question of recommendation also received very high approval ratings. The weekly workload reported by the participants was very heterogeneous (mean=2.4 hours; SD=1.1). For some participants, the use of the learning platform was not intuitive and group collaboration was somewhat faltering.

**Conclusion: **The experiences made show that courses on medical didactics can be implemented online and are gladly accepted by the participants. Based on the experience gained, online seminars or blended learning formats will certainly continue to be part of the FAM course program in the future.

## Zusammenfassung

**Zielsetzung: **Aufgrund des Verbots von Präsenzveranstaltungen im Zuge der Corona-Pandemie wurde das Seminar „Schriftliche Prüfungen“ der Frankfurter Arbeitsstelle für Medizindidaktik (FAM) in ein asynchrones Online-Seminar überführt. Dieses Pilotprojekt untersuchte, wie ein solches Format von den Teilnehmenden angenommen und bewertet wird.

**Methodik:** Es wurde ein forumsbasiertes Online-Format mit Gruppen- und Einzelaufgaben gewählt, das didaktisch nach dem problemorientierten Design nach Reinmann und Mandl gestaltet wurde.

**Ergebnisse: **Am Seminar haben 14 Personen teilgenommen, von denen sich 13 an der Evaluation beteiligten. Die Gesamtbewertung lag, mit einer Ausnahme, bei einer Note von 2 (und besser). Auch die drei Items „Praxisrelevanz“, „subjektiver Lernerfolg“ sowie der Frage nach der Weiterempfehlung erhielten sehr hohe Zustimmungswerte. Der von den Teilnehmenden angegebene wöchentliche Arbeitsaufwand war sehr heterogen (MW=2,4 Stunden; SD=1,1). Für einige Teilnehmende war die Bedienung der Lernplattform nicht intuitiv und stellenweise lief die Zusammenarbeit in der Gruppe etwas stockend.

**Schlussfolgerung: **Die gemachten Erfahrungen zeigen, dass medizindidaktische Fortbildungen grundsätzlich auch gut online umsetzbar sind und von den Teilnehmenden auch gerne angenommen werden. Aufgrund der gemachten Erfahrungen werden Online-Seminare oder auch Blended-Learning-Formate mit Sicherheit in Zukunft weiterhin zum Kursprogramm der FAM gehören.

## 1. Introduction

Due to the corona pandemic, all forms of classroom teaching were banned at the Goethe University Frankfurt from mid-March 2020 onwards, which meant that online offerings had to be developed within a short time as a replacement for the further training of teachers in medical didactics. Against this background, the transfer of an already existing course of the Frankfurter Arbeitsstelle für Medizindidaktik (FAM) into an online format is described, focusing on the didactic aspects.

## 2. Project description

The starting point was the already existing 1.5-day course “Written Examinations”, which was converted into an asynchronous, forum-based online seminar with group and individual tasks on the Moodle learning platform. This was intended to achieve greater time flexibility for the learners compared to synchronous formats. The seminar lasted six weeks and every week (except the last) there was a new task. Most tasks had to be worked on together in fixed groups, others had to be worked on alone (see attachment 1 ).

The didactic design was based on the model of problem-oriented learning by Reinmann and Mandl, whose four characteristic elements were implemented as follows.

### 2.1. Authenticity and application

The model requires the active participation of the learners through authentic problems with a high (subjective) relevance and a high reality content [5]. To achieve this, a framework story was presented that runs through the individual thematic blocks (see attachment 1 ). 

#### 2.2. Multiple contexts and perspectives

Learning in different contexts [2] and under multiple perspectives [5] is of great importance for the learning and later transfer of what has been learned. For example, the presented story has encouraged participants to consider different perspectives on the need for examinations. Dealing with exam questions from other disciplines also brought new contexts and different perspectives.

#### 2.3. Social learning arrangements

Working together and the associated mutual exchange is a further component of problem-oriented learning [5]. The work in this seminar took place almost exclusively in groups of three to four people. The group work and identity were promoted through measures such as self-chosen group names and participant profiles with photos, which also reduce the lack of social presence [1]. In addition, seminar rules were established at the beginning regarding cooperation and expected activity. 

#### 2.4. Instructional support

Learning without instruction or feedback is rarely successful and often leads to overstraining the learners [2], [5]. In addition to the clear instructions in the individual tasks, the information needed to complete the tasks was either provided or had to be acquired by the participants themselves. Each group promptly received written feedback from the instructor for each solution submitted. At the end of the seminar there was a video conference to clarify any remaining questions. 

## 3. Results

The slightly modified evaluation form of the FAM was used for evaluation. A total of 13 of the 14 participants filled out the evaluation form. The overall evaluation showed a very positive picture (see table 1 (Tab. 1)).

**Figure 1:**
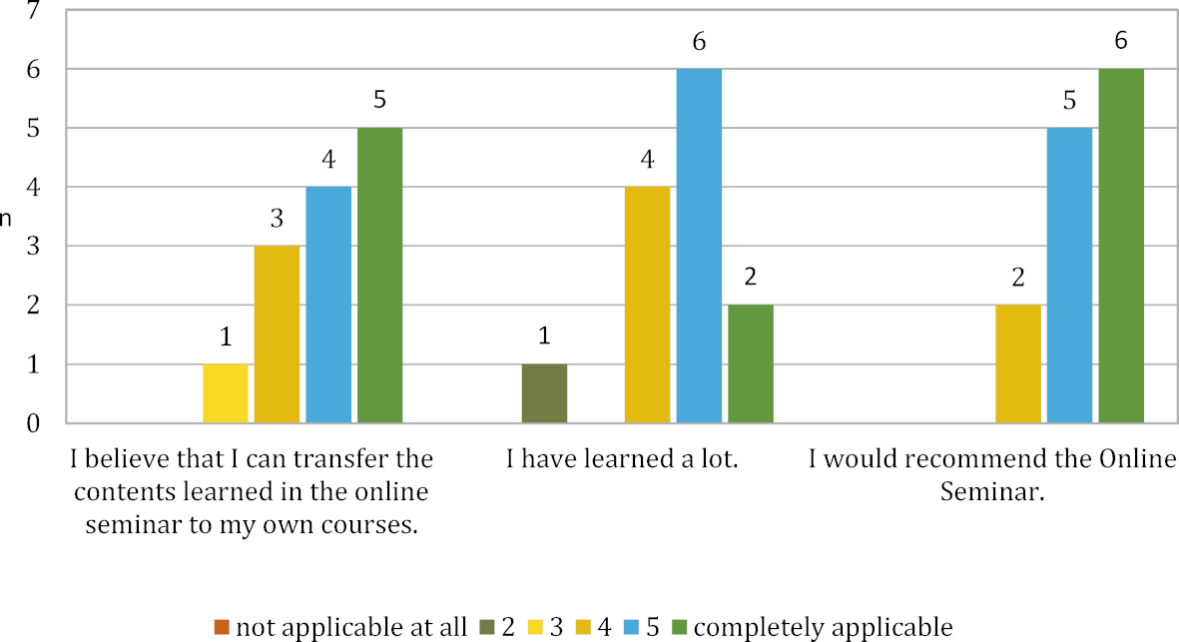
Selected evaluation results (n=13)

With regard to the three items “practical relevance”, “subjective learning success” and the question of recommendation, the result is similarly good (see figure 1 (Fig. 1)).

Two people criticized the group work or the exchange with the other seminar participants. In contrast, three persons explicitly emphasized the group work as positive in the free text answers.

Other aspects mentioned as positive were the supervision and feedback by the instructor (n=4) and the possibility to organise the time individually (n=2). The usability of the learning platform was rated slightly worse. At least four participants stated that they had had difficulties in this respect.

When the seminar was planned, the actual workload for the participants could only be estimated, which is why this was also queried (see table 2 (Tab. 2)).

## 4. Discussion

The following elements are important for a successful online seminar based on group work: creating both social presence and a sense of responsibility of the individual participants towards the group as well as continuous feedback. The evaluation results show that this was achieved well: on the one hand through the seminar rules and on the other hand through individual feedback after each task. Writing down this feedback took considerably more time than verbal feedback. While in a face-to-face seminar with 24 teaching units the work of the lecturer is concentrated on one and a half days (plus feedback for the follow-up tasks), the supervision of an online seminar of this form requires, in addition to the more elaborate feedback, a constant „online presence“ of the seminar leader, also to be able to react quickly to questions or problems. How much additional effort is required depends very much on the individual participants and the respective group composition. 

The seminar was planned with a scope of 24 units, which corresponds to three hours per week. With an average of almost two and a half hours per week, this goal was only just missed. However, it should also be considered that three persons completed the seminar, who only invested one to less than two hours per week. A look at face-to-face seminars, however, shows that here too the amount of work varies greatly both in the attendance phases and in the follow-up, and there are participants who tend to proceed according to the minimal principle. Accordingly, the degree of communication in the forums varied greatly depending on the motivation of the participants.

Since there were also participants who complained about the lack of social presence, a short initial video conference was scheduled for the second round of the course, in which the participants could introduce themselves to each other and in which the most important functions of the learning platform were also demonstrated.

## 5. Conclusion

In its form, the online seminar presented here offers a good opportunity to provide at least a limited range of courses in the current crisis situation, in which many of the other courses cannot be held. The experiences made so far show that this is also gladly accepted by the participants and thus online seminars like this one at the FAM will certainly continue to be offered either in this form or as blended learning seminar.

The establishment of online seminars is currently still hindered by the regulations on the mutual recognition of medical didactic offers of the MedizinDidaktikNetz Deutschland [4]. In these, attendance times of at least 50% of the total scope of the basic qualification (MQ 1) are required. If attendance times are replaced by online elements, this required minimum can sometimes no longer be achieved. It needs to be discussed how this criterion could be further developed with regard to asynchronous online training, for example, to the effect that >50% of the course time must be spent working with other participants in order to ensure interaction similar to that in a normal course.

## Competing interests

The authors declare that they have no competing interests. 

## Supplementary Material

Tasks of the online seminar

## Figures and Tables

**Table 1:**
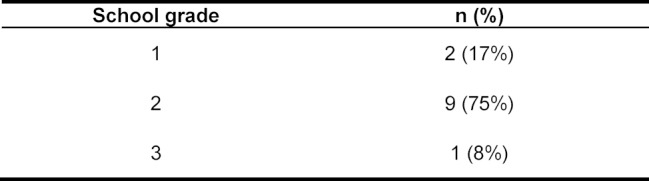
Final evaluation by German school grades (n=12)

**Table 2:**
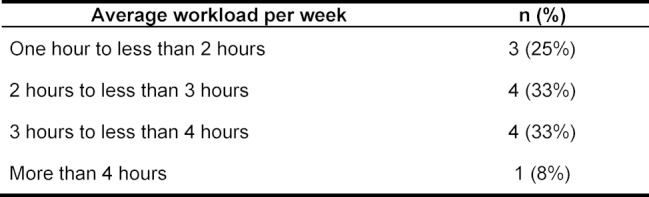
Average workload per week (n=12, Mean=2.4 hrs, SD 1.1)
