# Supplementary material for: Medical didactics during the pandemic: the asynchronous online seminar “Written Examinations” of the Frankfurter Arbeitsstelle für Medizindidaktik
Source: GMS J Med Educ. 2021 Jan 28;38(1):Doc18. doi: 10.3205/zma001414 (PMC7899102; doi:10.3205/zma001414)
Supplement: Tasks of the online seminar [file JME-38-1-18-s-001.pdf]

Attachment 1: Tasks of the online seminar

<table>
<tr><th>Time</th><th>Tasks</th></tr>
<tr><td>Week 1</td><td>Dear participants,

here are your tasks to be completed within one week:

• Complete your profile with a profile picture and other personal information.
• Read and comment on the seminar rules.
• To get started, answer a few questions about your experience with exams [these questions have been asked and answered in a separate forum].
  o What was the worst exam or exam situation for you? Why?
  o What exams are currently available in your department?
  o How are you involved in them or how would you like to be involved in the future?
• Assign yourself into a group. For easier coordination, it can be helpful to get together with colleagues from your own department. If you do not have any preferences, I will be happy to make the division for you. The group size should be 3 to 4 people.
• Think about a group name together in the forum “Your group names”. Please let me know the name (e.g. by e-mail or via message on the learning platform). You can create a new topic in the forum "Your group names" that is only visible to your group by clicking on “Add new topic”.</td></tr>
<tr><td>Week 2</td><td>Dear participants,

the new dean Prof. Dream will take up his duties next month and some of his plans have already been revealed. Prof. Dream is no friend of exams and now he plans to ban all exams at the faculty, so that only the state exams remain.

From my point of view this is not a good idea. In order to possibly change the dean's mind, I need your support:

Your task is therefore to discuss in your respective group forums (via “Add new topic”) what reasons there might be to object to Prof. Dream. As a conclusion of your discussion, formulate a written plea for the retention of examinations by <date>, which I can read out in the next Study Committee.

I am looking forward to your results!</td></tr>
<tr><td>Week 3</td><td>Dear Sir or Madam,

Your remarks on the importance of examinations during studies convinced me to stick to examinations after all.

In addition, I asked around with colleagues in other faculties and asked how examinations are implemented here. Here are the answers:</td></tr>
</table>

Attachment 1 to: Kollewe T, Ochsendorf F. *Medical didactics during the pandemic: the asynchronous online seminar “Written Examinations” of the Frankfurter Arbeitsstelle für Medizindidaktik.* GMS J Med Educ. 2021;38(1):Doc18. DOI: 10.3205/zma001414

	• At the university “Oralia” there is a course on pathophysiology in the 4th preclinical semester. The course with 60 students runs over 2 semesters and at the end there is a written exam. Now there is an oral intermediate examination in addition to the end of the 1st semester.<br>• The university “Textor” describes a lecture series in the 4th clinical semester on paediatrics. The semester has a strength of 250 students. At the end an exam with MC questions is written.<br>• The colleague at the university “Quaestio aperta” told me about an internal medicine course in the 5th clinical semester. The semester has a strength of 150 students and it is important to him to introduce an additional alternative examination form in addition to the state IMPP examination, which is why a written exam with open questions is written at the end.<br>• From the university “Do City” I was told about a course to learn examination techniques in the 1st clinical semester. The groups consist of 4-5 students each. At the end of the course there is a practical examination.<br><br>Unfortunately, I do not know enough and need your expertise:<br><br>Why did the locations decide on the respective examination form and what do you as a group see as strengths and weaknesses of the individual forms?<br><br>I look forward to your group answer by <date>.<br><br>With kind regards<br><br>Prof. Dream
Week 4	In this week, the following individual tasks had to be completed. A new task was only activated after completing the previous one:<br>• UTC-Test (UTC is the abbreviation for “Use The Cues”. Each of the seven [...] questions contain a typical formal or linguistic solution hint. [3]).<br>• Presentation on important aspects of question formulation (according to [2])<br>• Own guideline-based creation of a case-based MC question (according to [2])
Week 5 + 6	Dear Sir or Madam,<br><br>I used the last week to look at some questions from the semester-end exams. In my opinion, there is still room for improvement. If we bundle your expertise, we'll soon have a nice catalogue of questions.<br><br>• Each of you please create four more questions in addition to the one already formulated last week. You will then upload your five questions to your group forum. Remember to also document the learning objectives to be examined.<br>• Afterwards you will evaluate at least five questions from your group members. You can use the checklist from last week as a guide, but you should not just answer the individual questions on the checklist with yes or no. Your decisions should also be justified.<br>• Use the reviews to optimise your own questions.

	• Once all questions have been created, reviewed and optimised, this group phase is complete.<br><br>I look forward to your results.<br><br>Kind regards<br><br>Prof. Dream
Always available	Dear Sir or Madam,<br>For your own course or subject, create a blueprint for a 30-question exam and upload it.<br><br>This task is preceded by an explanation and an example of a Blueprint.
In between	In addition to feedback on the submitted solutions, the participants were given advice and literature on thematically appropriate aspects, such as the distinction between summative and formative tests or test quality criteria.
